# Hidden State Inference or Continuous Belief Updating during a Dynamic Visuomotor Skill

**DOI:** 10.1523/JNEUROSCI.1285-25.2025

**Published:** 2025-12-23

**Authors:** David J. Harris, Tom Arthur

**Affiliations:** School of Public Health and Sport Sciences, Medical School, University of Exeter, Exeter EX1 2LU, United Kingdom

**Keywords:** Bayesian inference, predictive processing, perception, sensorimotor, virtual reality

## Abstract

Adaptive sensorimotor behavior requires individuals to flexibly update their beliefs in response to changes in environmental context. Theories of predictive processing propose that such flexibility arises from hierarchical inference, where higher-level beliefs about hidden states shape lower-level perceptual and motor predictions. Here, we test whether behavior in a naturalistic interception task is better explained by continuous belief updating or by more abrupt shifts driven by state inference, consistent with hierarchical Bayesian learning. Twenty-three participants (9 females and 23 males) completed 160 trials of a VR-based interceptive task across two sessions. Participants attempted to return balls with varying bounciness, probabilistically cued by ball color and a contextual cue (court wall color) that reversed periodically. Gaze location prior to bounce was used as an index of perceptual inference and modeled using Bayesian and reinforcement learning frameworks. Continuous belief updating Bayesian models (the hierarchical Gaussian filter) outperformed associative learning models in predicting participant behavior. In addition, continuous belief updating, rather than discrete state, better explained participants’ gaze behavior, despite the contextual state-inference model better anticipating the true structure of the task. Overall, while participants updated beliefs in a Bayesian manner, their behavior fell short of normative optimality, possibly due to limits on causal inference and model construction. This may reflect the specific demands of dynamic sensorimotor control, where the brain prioritizes flexible, real-time updating over explicit structural inference to support fluid, adaptive action.

## Significance Statement

How does the brain adapt to changing environments during fast-paced actions like intercepting a bouncing ball? Theories of predictive processing suggest that the brain builds internal models of the world and updates them when contexts shift. Using a virtual reality task and eye tracking, we examined how people adjust their expectations in a dynamic sensorimotor setting. Our results show that although participants updated beliefs in a Bayesian manner, they did so continuously rather than by inferring sudden changes in context. This reveals a gap between idealized models of cognition and how the brain handles real-world demands. The findings offer insight into the flexible yet resource-limited nature of human learning and have implications for models of perception, action, and decision-making.

## Introduction

This paper explores the degree to which individuals utilize contextual information to infer higher-level “hidden states” that subsequently exert top–down influences on perception and action. The ability to flexibly adapt sensorimotor skills is critical for responding effectively to the dynamic and uncertain demands of real-world environments (Cisek and Kalaska, 2010; Clark, 2015). However, the neurocomputational mechanisms through which higher-level beliefs shape moment-to-moment perception and action behaviors remains unclear. Influential Bayesian models propose that adaptive human perception and learning arise from a hierarchical cortical organization, where higher-level beliefs shape lower-level predictions (Felleman and Van Essen, 1991; Heilbron and Meyniel, 2019; Yon and Frith, 2021). In this framework, prediction errors update inaccurate beliefs, while higher-level beliefs modulate the precision of these errors, controlling how strongly sensory input influences updates. This dynamic weighting allows the brain to balance stability and flexibility, though studies of naturalistic sensorimotor behavior are needed to test these computational accounts (Fooken et al., 2023).

**Figure 1.**
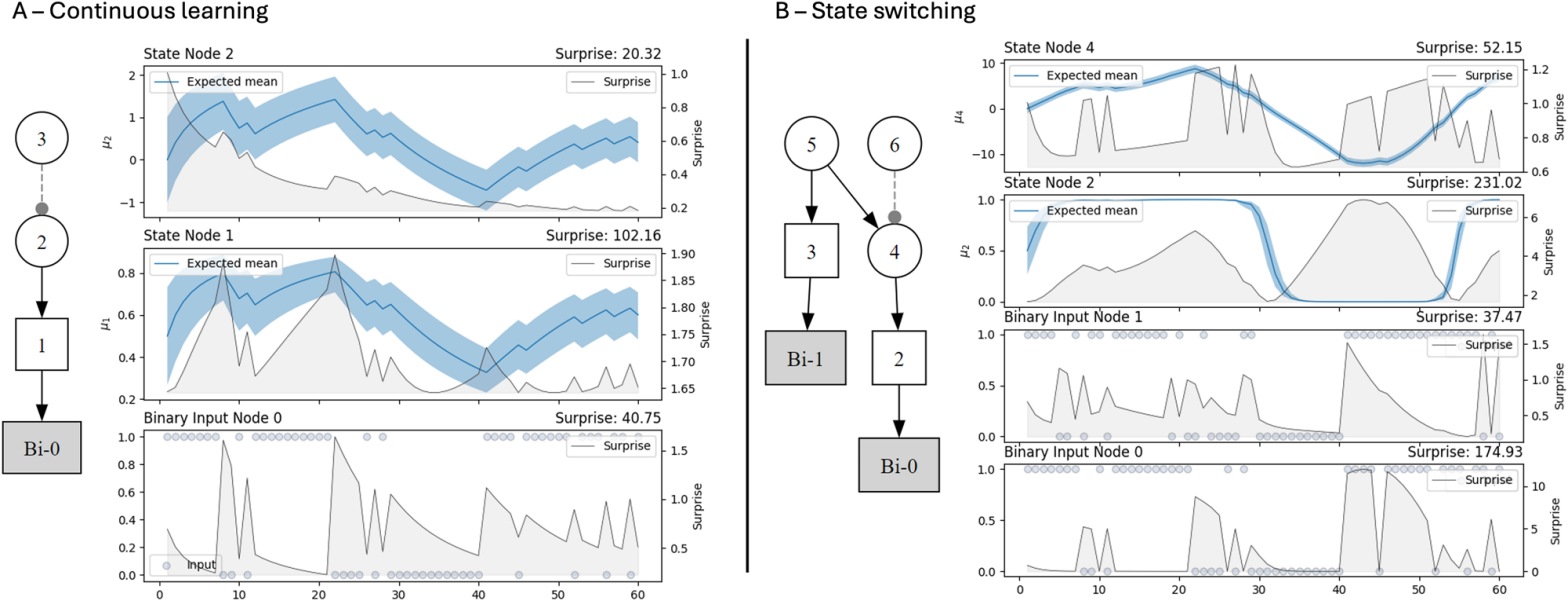
Simulations of continuous learning and state-switching modes of learning in a Bayesian network. Identical trial orders were entered at node Bi-0, consisting of a context switch from 87/13 to 13/87 and back again (bottom panel for each model). In ***A***, this quantity is continuously updated, gradually shifting from one outcome to the other. In ***B***, the agent also receives a context cue at Bi-1 (which is less reliable than at Bi-0), which updates a node (5) which is a value parent of 4. Such that 4 is not only updated with incoming information at Bi-0, but its predictions are partly set by 5. The result is clearer belief switching from Outcome 1 to Outcome 0 (see State Node 2 in panel ***B***), compared with continuous updating in ***A***. An interesting feature of the current model is that the switches do not happen immediately; instead the agent retains a belief in Outcome 1 until sufficient evidence has accrued at state Node 4, and then the belief performs an abrupt switch.

Studies of sensorimotor behavior have emphasized how “contextual cues,” which provide information about the setting or circumstances in which an event takes place, shape skilled performance (McRobert et al., 2011; Gredin et al., 2018; Runswick et al., 2018). Here, “context” can refer to directly perceivable aspects of the environment or to latent, unobserved (i.e., hidden) states that have to be inferred based on changing outcomes (e.g., from a predictable to an unpredictable context; Schuck et al., 2016; Vaidya et al., 2021; Zika et al., 2023). In anxiety disorders, for example, returning to a context linked to past aversive outcomes can trigger sudden behavioral shifts (Zika et al., 2023). Such state-dependent learning contrasts with the gradual updates described by classical associative learning theories (Rescorla and Wagner, 1972). Specifically, in gradual learning [e.g., Kalman filter, Rescorla–Wagner (R–W) models], the individual revises their expectations incrementally, adjusting them trial by trial and continuously refining the previous estimate with each new experience. This is illustrated in Figure 1*A*, which shows a simulation of a Bayesian agent (represented by the factor graph) gradually updating their beliefs about the true state of the world (see state Node 1 after the inputs switch from 1 to 0 s and vice versa). Such iterative updating necessitates a continuous learning process, albeit one that could still adapt quickly when circumstances change. Conversely, in state-dependent learning (Fig. 1*B*), the agent simultaneously infers a higher-order hidden state of the world (or “context”), and beliefs about this state exert a top–down influence on lower-level representations, resulting in more distinct shifts in predictions, known as state switches (note the abrupt transitions in Fig. 1*B* driven by inferences about context in Nodes 3 and 5). From a theoretical perspective, state-switching behaviors are indicative of a hierarchical organization of neural representations and therefore align closely with the principles of predictive processing and active inference (Friston et al., 2009, 2016).

In the present work, we investigate this issue by examining whether the oculomotor behavior of participants in an interceptive task is more consistent with the continuous learning or state-switching simulations displayed in Figure 1. Adopting a computational modeling approach, we firstly hypothesized that participants’ gaze patterns would be better explained by Bayesian learning models than associative learning models, given the growing body of evidence that perception and action are linked to approximations of Bayes-optimal computations (Körding and Wolpert, 2004; Arthur and Harris, 2021; Hipólito and Kirchhoff, 2023). Secondly, we hypothesized that eye movements would be better explained by a context learning model (enabling state-switching dynamics) than a continuous learning model, as predicted by hierarchical theories of learning (Felleman and Van Essen, 1991; Iglesias et al., 2013; Heilbron and Meyniel, 2019). By probing the nature of these learning mechanisms in a dynamic visuomotor task, this study aims to provide a novel test of state-dependent learning, bridging the gap between computational theories of inference and embodied sensorimotor behavior.

## Materials and Methods

### Participants

We employed an opportunity sample of individuals, recruited from the student population at the host university. Twenty-three participants (nine females) took part in the experiment, all of which were included in the final analysis. Participants had a mean age of 21.3 years (SD, 0.86; range 20–23). Of the 23 participants, 12 reported having used VR before, but none reported using it regularly. Participants had no previous adverse reactions to VR or history of motion sickness, nd were not red/green colorblind (all self-reported). Participants were provided with details of the study and gave written informed consent on the first day of the two testing visits. Ethical approval was obtained from the departmental Ethics Committee prior to data collection (REF: 5487677) which took place between January 2024 and May 2025.

This study employed a cross-sectional design to analyze a targeted subset of training data from participants involved in a larger research project on interceptive performance (10.17605/OSF.IO/APU3R). Consequently, the sample size (*N* = 23) could not be determined a priori. Following Schönbrodt and Wagenmakers (2018), we assessed evidential sensitivity post hoc by examining whether the primary model comparison analyses yielded conclusive Bayes factors (BF₁₀ > 3 or <1/3; Kass and Raftery, 1995). All three of the main comparisons met or exceeded these thresholds, indicating that the data were sufficiently informative for the hypotheses tested and that increasing the sample size would likely not alter the qualitative conclusions.

### Experimental design

**Figure 2.**
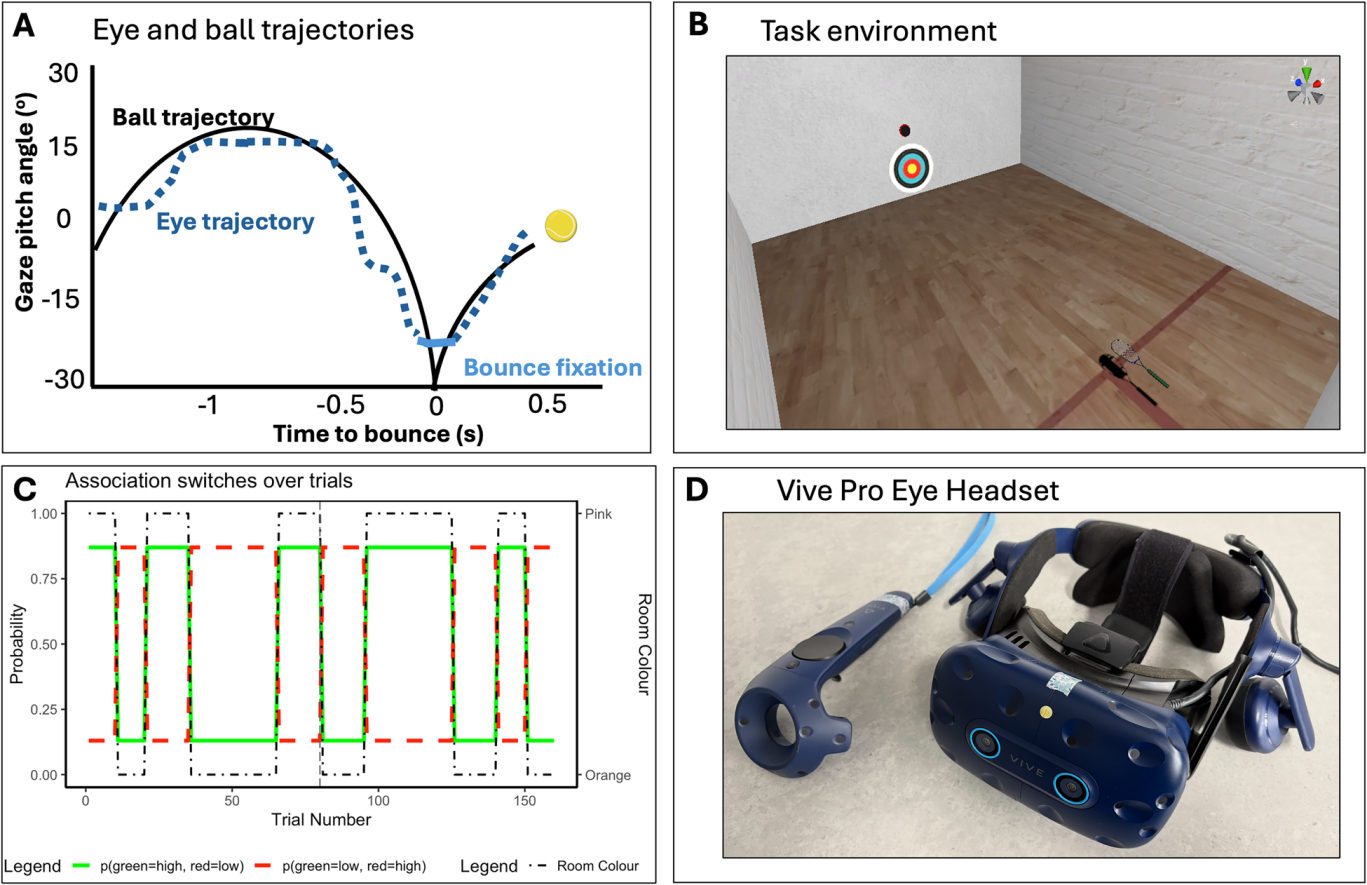
The task environment and trial probabilities. Panel ***A*** illustrates the typical eye gaze strategy during this task. Panel ***B*** shows the VR squash court environment. Panel ***C*** shows the reversal learning paradigm: pink and orange lines indicate the switching probabilistic relationships over trials; vertical dotted lines indicate when the participant had breaks. This was one of the eight possible orders which were counterbalanced across participants.

Participants completed 160 trials of a virtual reality interceptive task (split evenly across two visits approximately a week apart) in which the color of the ball and its subsequent bounciness exhibited probabilistic, context-sensitive relationships that changed over time (Fig. 2*C*).

### Apparatus and stimuli

The experimental task consisted of a virtual reality simulation of an indoor squash court [previously used in Arthur et al. (2021); D. J. Harris et al. (2023)], developed using the gaming engine Unity (Unity Technologies). This environment was displayed using an HTC Vive Pro Eye headset (HTC; Fig. 2*D*), a high-precision, consumer-grade VR system with 110° field of view (accuracy 1.5 cm, jitter 0.5 mm, latency 22 ms; Niehorster et al., 2017). The Unity environment recorded movements of the headset and hand controller at 90 Hz. The headset included a Tobii eye-tracking system, which employs binocular dark pupil tracking to monitor gaze at 120 Hz (spatial accuracy 0.5–1.1°; latency 10 ms). To establish tracking accuracy, we calibrated gaze over five virtual locations at the start of each experimental session and upon any obvious displacement of the headset during trials.

At the start of each trial, a ball was launched from the front of the virtual squash court (Fig. 2*B*) from a height of 2 m, which was just above an aiming target that consisted of a series of concentric circles. Participants stood in the center of the court (15 × 15 m), ∼9 m from the front wall [as in Diaz et al. (2013a)]. Participants attempted to hit the projected balls, which looked like real tennis balls (5.7 cm in diameter), back toward the target using a virtual racquet that was animated in the virtual world by tracking the handheld VR controller. The virtual racquet was 0.6 × 0.3 × 0.01 m, but the collision area associated with it was exaggerated by 20 cm to enhance the detection of ball-to-racquet collisions.

Trial orders were designed to probe how participants learned associations between ball color and bounciness. On each trial, an oncoming tennis ball—either red or green—had one of two distinct elasticity profiles, creating different levels of bounciness. The two bounciness profiles were highly distinct; low trials were hit below waist height and high trials were hit around head height (in relation to the average height of an adult in the United Kingdom: 1.70 m), creating a demand to accurately anticipate bounciness as much as possible. Trials were organized into blocks with a probabilistic relationship between ball color and bounciness: either green balls were bouncy and red were not (with an 87% probability) or the reverse. Periodic switches occurred throughout the experiment, altering which color was associated with high bounciness. Specifically, there were four switches in each of the two testing sessions (Fig. 2*C*). Each switch was accompanied by a change in the color of the court's side walls (pink or orange), serving as an associative contextual cue. Participants were not explicitly informed about the relationship between room and ball colors and bounciness outcomes, but it was indicated to them that the colors potentially had some relevance to the task (see Supplementary File 1 available on https://osf.io/yq2bv/files). This setup allowed us to assess how participants learned the color–bounciness associations, whether room color changes would be encoded within higher-level contextual beliefs to influence this learning, and the resulting effects on participants’ perception and action.

### Measures

#### Gaze pitch angle

**Figure 3.**
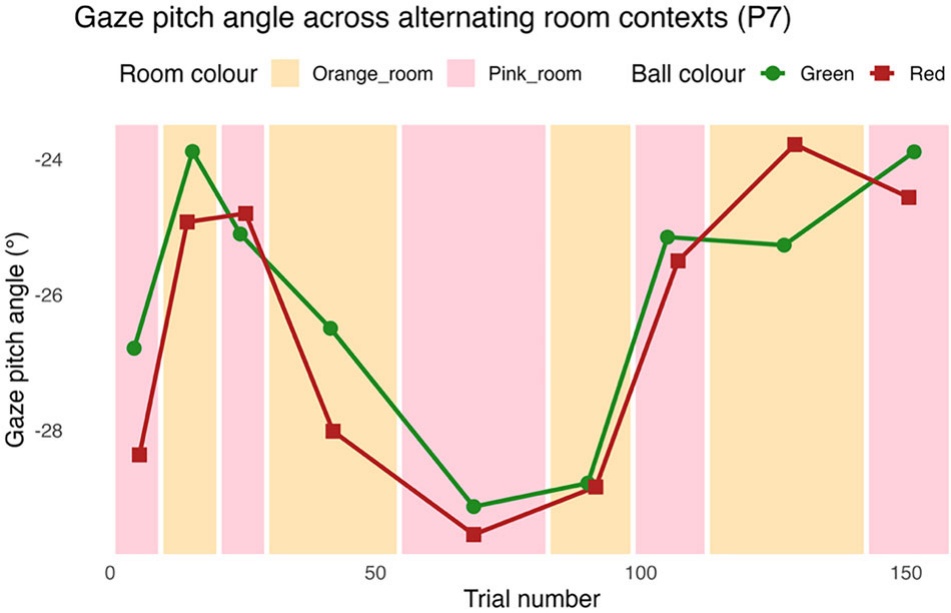
Context-dependent changes in the gaze pitch angle for a single participant. Lines show the mean gaze pitch angle for green and red balls across the two room colors. Means are computed per block of consecutive trials in the same room. The room/ball combinations are such that fixations would be expected to be higher for green balls in the pink room and higher for red balls in the orange room.

Oculomotor behavior in this task is known to be sensitive to expectations about bounciness (Diaz et al., 2013a; Arthur et al., 2021; Arthur and Harris, 2021). Individuals generally direct a single fixation to a location a few degrees above the bounce position of an oncoming ball (Diaz et al., 2013b; Mann et al., 2019; Fig. 2*A*). Crucially, it is known that when the elasticity of the ball is changed, individuals tend to adjust the height of the prebounce fixation to account for the ball's postbounce trajectory (Diaz et al., 2013a), illustrating that gaze is guided by prior experience. The spatial position of this fixation (referred to here as the “gaze pitch angle”) is therefore linked to beliefs about likely ball trajectories, with fixations directed to a higher location when higher bounces are expected. The rate at which the location of this fixation is adjusted over time is also dependent on the inferred changeability of the environment (Arthur and Harris, 2021). As the fixation occurs before the bounce is observed, the fixation location is driven by an agent's prior expectations about ball elasticity and provides an indicator of the evolution of beliefs over time. This variable therefore allows us to model the underpinning beliefs driving oculomotor control (i.e., active inference) and whether these exhibit state-switching dynamics. A plot of the gaze pitch angel across the block reversals is shown in Figure 3.

### Procedure

Data collection took place over two visits lasting ∼45 min each (1.5 h in total). Upon arrival at the lab, participants had the experimental procedures explained to them and provided written informed consent. Participants viewed a standardized video outlining the study's purpose, followed by a verbal explanation from the researcher. Participants were fitted with an HTC Vive Pro Eye VR headset and underwent a calibration process to ensure proper fit, eye position alignment, and accurate eye movement detection. Participants were placed in the virtual environment (Fig. 2*B*) and stood in the center of the 15 × 15 m court, 9 m from the front wall [as in Arthur et al. (2021)]. On each trial, a virtual tennis ball (5.7 cm in diameter) was projected from the front of the court from a height of 2 m, directly down the center line, and bounced 3.5 m in front of the prescribed starting position on the “short line.” Ball release on each trial was cued by an auditory tone (three beeps). Each ball followed an identical prebounce trajectory and speed (vertical speed, −9 m/s at time of bounce), which were both consistent with the effects of gravity (−9.8 m/s^2^). Just below the ball release point was an aiming target that consisted of a series of concentric circles. Participants were instructed to return the ball back toward the center of the target using the virtual racquet (0.6 × 0.3 × 0.01 m), which was animated by tracking the handheld VR controller.

A familiarization set of six yellow balls (designed to avoid color learning) preceded the main experiment. These balls alternated between the two elasticity levels: “bouncy” and “nonbouncy.” Participants then completed 80 trials of the color–bounce association trials on each of the visits (160 in total). Participants were allowed breaks when needed. Previous studies have shown that probabilistic relationships can be learn in relatively few trials (10–20; Berniker et al., 2010), so the reversals in the color–bounce relationship were programmed after 20–30 trials. Trial orders (and all other supplementary files) can be seen in the online supplementary files (https://osf.io/yq2bv/files).

### Data processing

A unit gaze direction vector was obtained from the HMD eye-tracking system, with its features defined in head-centered, egocentric coordinates. This gaze vector, along with the ball's position relative to the head, was plotted in a 2D space to create angular orientation metrics within the environment. Each trial was segmented from the moment the ball was released to the frame corresponding to ball contact. Trials with >20% missing data or instances where eye tracking was lost for over 100 ms were excluded from analysis. Gaze coordinates were processed using a three-frame median filter, followed by a 15 Hz second-order Butterworth filter (Cesqui et al., 2013; Fooken and Spering, 2020). A spatial dispersion algorithm was used to identify gaze fixations (Krassanakis et al., 2014), which were defined as periods when the point of gaze remained within 3° of the visual angle for at least 100 ms (Salvucci and Goldberg, 2000). Fixation position at the moment of ball bounce was recorded as the gaze-head pitch angle. Data points exceeding 3.29 standard deviations from the mean (*p* < 0.001) were classified as outliers, and participants with >15% of data marked as missing or outliers were removed from analysis. All supplementary files, including model checking analyses, can be found online: https://osf.io/yq2bv/files.

### Computational modeling

We sought to examine the perceptual inference processes of participants by fitting learning models to the gaze pitch angle variable. In several previous papers, we have shown that Bayesian inference provides a compelling explanation of the trial-to-trial updating of eye movements in this and closely related tasks (Arthur and Harris, 2021; Arthur et al., 2023; D. Harris et al., 2023). The present task, however, presented a more complex inference challenge as participants were not simply inferring the likely postbounce ball trajectory but the shifting probabilistic associations between color cues and subsequent outcomes. We, therefore, sought to firstly test whether Bayesian inference models provided the best explanation of behavior in this task. Secondly, we examined whether a state-switching or continuous learning model provided a better explanation of participant behavior. Finally, we examined whether the computational parameters that characterized each individual's perceptual inference were predictive of their task performance.

#### Model comparison

To determine whether hierarchical Bayesian models with dynamic learning rates provided a good explanation of learning in this task or if simpler explanations could be provided, we compared two traditional reinforcement learning models with two-, three-, and four-level variants of a Bayesian learning model called the hierarchical Gaussian filter (HGF; Mathys et al., 2011, 2014). Each of these models is described in more detail below (for further detail, see Supplementary File 3 available on https://osf.io/yq2bv/files).

*R–W and Sutton K1 associative learning*. Reinforcement learning models assume that agents use prediction errors from previous trials to update beliefs to maximize the probability of future rewards (Rescorla and Wagner, 1972). In R–W learning, predictions about a value (*v*) are updated over trials (*k*) in proportion to the size of the preceding prediction error (*δ*) and a stable learning rate scalar (*α*):

$$\Delta v^{k}\;\propto \;\alpha \delta^{k}.$$

The Sutton K1 model—sometimes described as a R–W–Pearce–Hall hybrid (Tzovara et al., 2018)—assumes the same general form but allows for variable learning rates that are scaled by recent prediction errors (Sutton, 1992), whereas the R–W model assumes fixed reward magnitudes. Specifically, the gain (*α^k^*) adapts over time according to the magnitude of recent errors as follows:

$$\Delta v^{k}\;\propto \alpha^{k}\delta^{k},$$

$$\alpha^{k+1}=\;\alpha^{k}+\eta (|\delta^{k}|-\;\alpha^{k}),$$

where *η* is a small positive constant controlling the rate of gain adaptation. Notably, both models differ from hierarchical Bayesian learning models (Mathys et al., 2014; Smith et al., 2022) as the impact of the prediction error is not dynamically adjusted based on higher-level beliefs or encoding of environmental volatility.

*HGF*. The HGF serves as a generative model, explaining how an agent receives a time series of inputs (*u*) and subsequently produces a series of responses (*y*; Mathys et al., 2011, 2014). Importantly, when both inputs and responses are known, the model allows for the estimation of perceptual and response parameters by inverting the model, enabling the extraction of participant-specific parameters and belief trajectories (Mathys et al., 2014). The HGF assumes that an agent is trying to learn about (i.e., infer on) a continuous uncertain quantity *x*, which shifts over time. A generic way of describing the evolution of the belief about this quantity is as a Gaussian random walk, which describes the evolution of a time series via a Gaussian probability distribution over *x* as follows:

$$x^{\left(k\right)}∼N(x^{\left(k-1\right)},\upsilon ),$$

where *x*^(*k*−1)^ is the mean of the distribution and *υ* is the variance at the preceding timepoint. The variance (or “tonic volatility”) parameter determines the rate at which the belief *x* evolves over time in light of new observations. Updates to beliefs proceed according to Bayes theorem, where the prior belief over *x* is updated with new observations, weighted by their precision.

In the two-level version of the HGF, variance in the uncertain quantity *x* is determined by the parameter *υ*, but uncertainty about *υ* may itself also vary. In such a case, it may be adaptive for the agent to change the rate at which they update their beliefs (Behrens et al., 2007). Bayesian models of learning assume that higher-level inferences about the state of the world can dynamically adapt learning rates in this way. Specifically, when the environment is seen to be changeable (volatile), it is often beneficial to update beliefs more quickly. Simple reinforcement learning models do not explicitly track environmental volatility in this way, although they can be adapted to be sensitive to past prediction errors. We can achieve this dynamic learning rate using the HGF quite simply by replacing the tonic volatility value *υ* with a second random walk function where the volatility value can itself shift over time. This can be extended to any number of hierarchical levels as follows:

$$x_{i}^{\left(k\right)}∼N(x_{i}^{\left(k-1\right)},f_{i}(x_{i+1})),\;i=1,\ldots ,\;n-1.$$

Here we implement two-, three-, and four-level versions of this model. A more technical and detailed description of the HGF is provided in Supplementary File 3 available on https://osf.io/yq2bv/files.

For this initial model comparison, the open-source software package TAPAS (available at http://www.translationalneuromodeling.org/tapas; Frässle et al., 2021) and the MATLAB version of the HGF toolbox (Mathys et al., 2011, 2014) were used for both model fitting and comparison. For all perceptual models, including the RL models, the likelihood of the observed responses was defined through a common sigmoid observation model, which provides a probabilistic mapping from predicted states to binary responses via a decision noise parameter. Observations (*u*) were recoded to correspond to *p*(bounce|color) (a similar approach to Iglesias et al., 2013), so that the state in which 87% of green balls bounced high and 87% of red balls bounced low was coded 1, and the opposite state was coded 0. This dual coding of observations captures the contrasting beliefs about the relationship between ball color and bounciness. Anticipatory eye positions (the gaze pitch angle) were then dichotomized to create the response variable *y*. Eye position was taken to indicate a shift toward an expectation of a lower bounce when there was >1 standard deviation shift in position downward (and vice versa for higher bounces). Dichotomizing eye position using this threshold approach aims to focus the analysis on substantial, behaviorally meaningful shifts in gaze, reducing the influence of minor, potentially noisy fluctuations, and is consistent with previous work (Arthur and Harris, 2021; Harris et al., 2022; Arthur et al., 2023).

All learning models contained free parameters that could vary to accommodate the observed data that we wished to estimate. These parameters were optimized using maximum a posteriori estimation to provide the highest likelihood of the data given the model and parameter values. For associative learning models, the free values were beliefs about *p*(bounce|color) and the learning rate, which were set at a neutral starting value and given wide variance. For unbounded parameters in the HGF models, we chose prior means that represented values under which an ideal Bayesian agent would experience the least surprise about its sensory inputs, based on running a simulation with the real sequences from the experiment. The priors were given a wide variance to make them relatively uninformative and allow for substantial individual differences in learning (Table 1).

**Table 1.** Prior means and variances of the perceptual models

Parameter	Prior mean^a^	Prior variance
*Two-, three-, and four-level HGF*		
*κ*^b^	1	0
*ω*	−5.6	8
*ϑ*	−4	0
*μ*_2_	0	8
*σ*_2_	0.1	1
*μ*_3_	1	8
*σ*_3_	1	1
*μ*_4_	1	8
*σ*_4_	1	1
*R–W and SK1 model*		
*α*	0.5	1
*v*	0.5	1
*Context switching*		
*κ*^b^	1	0
*ω*	−5.6	8
*ϑ*	−4	0
*μ*_2_	0	8
*σ*_2_	0.1	1
*μ*_3_	0	8
*σ*_3_	0.1	1
*μ*_4_	1	8
*σ*_4_	1	1
*μ*_5_	1	8
*σ*_5_	1	1
*μ*_6_	1	8
*σ*_6_	1	1

a The HGF class prior means were determined by running a Bayes-optimal simulation of the task (where the variances were set wide to account for individual differences) and taking the resultant posterior means as starting values here (Mathys et al., 2011).

b Kappa, which allows a variable strength of coupling between levels, was fixed to reduce model complexity in light of the relatively few trials.

#### State-switching versus continuous learning

To foreshadow the results, the three-level Bayesian model was found to better account for the observed data than the other HGF variants or associative learning models. We therefore proceeded to compare this simple Bayesian learner with a more complex state-inference model to determine whether eye movements were indicative of continuous learning or state switching. To build this more complex model, we used PyHGF, an extended neural network library in Python that allows the development of more complex node structures within the HGF framework (Weber et al., 2023; Legrand et al., 2024).

The state-switching model (Fig. 1, right) effectively consisted of two arms, a ball color arm and a context arm. The first arm was the same three-level HGF inferring on the true color–bounciness association. The second arm featured a second input node receiving the room color observation. This input was connected to a binary state node (3) and a Gaussian state node (5) tracking this belief. This Gaussian node (5) was, however, also a parent of the Gaussian state node (4) in the ball color arm. The inferred context state therefore also set the belief about color and bounciness in a top–down fashion as described in predictive coding and active inference models. This version of the model learned the state identity and state–outcome probabilities through trial-by-trial updating, in the same way as the standard three-level HGF.

The two models were fit to the behavioral data using Hamiltonian Monte Carlo sampling in PyMC (Patil, Huard, and Fonnesbeck, 2010). A custom response function was defined for the models, such that three parameters (autoconnection strength at Nodes 2 and 4 and tonic volatility at Node 4) were optimized through gradient descent on the surprise of participant behaviors (analysis scripts are available on the OSF: https://osf.io/yq2bv/files). These three parameters were chosen to allow the rate at which beliefs were updated to vary by participant to capture individual differences in learning and optimize models fits. “Tonic volatility” is a node-level parameter that sets the baseline variance of the state transitions at a given hierarchical level, typically influencing how rapidly beliefs about that level are updated. It defines the constant component of volatility in the generative model, independent of phasic (trial-by-trial) volatility estimates propagated from higher levels. Tonic volatility at Level 4 was set as a free parameter (with Gaussian prior; mean = −5.0; SD = 2.0). The “autoconnection strength” parameter (*λ* ∈ [0,1]) refers to how strongly the previous state of a node influences its prediction of the next state. Here we use it as an index of the speed of belief updating (effectively a trait level learning rate)—i.e., how stable or changeable beliefs are. Higher values indicate slower belief updating (more constrained by prior state) and vice versa. If *λ* = 0, the node is not influenced by its own mean, merely its parents. *λ* at Levels 2 and 4 were set as free parameters (with a uniform [1,1] beta prior).

Surprise was then calculated as a linear function of the expected probability of the ball–color relationships at each timepoint in the model (Node 1 in the HGF3 and Node 2 in the context switching model). The surprise elicited by a binary observation x under the expected probability $\hat{\mu }$ is given as follows:

$$\{\begin{array}{cc} -\log\;(\hat{\mu }), & \mathrm{if}\;x=1\\ -\log\;(1-\hat{\mu }), & if\;x=0 \end{array}.$$

After models were fit to each participant's eye movements (effectively recovering the parameters that generated the behaviors), the optimized parameters were recorded. The two models were then run forward using the optimized parameters to determine the binary surprise given participants’ observed actions. We then compared the binary surprise generated by the two candidate models. Here, we used the binary surprise values, since we were interested in simply determining the most accurate description of the behavior rather than penalizing model complexity (as metrics like AIC and BIC do).

#### Relationship between task execution and model parameters

Finally, we examined the relationship between the estimated computational parameters for each individual and their overall task performance. We investigated the tonic volatility and autoconnection parameters that were optimized during model fitting, plus mean and precision of beliefs at Level 4 of the model and volatility beliefs. These are summarized in Table 2.

**Table 2.** Parameters recorded from the model

Notation	Name	Function
*ω_n*	Tonic volatility at node *n*	Static parameter governing how much the node's beliefs are expected to fluctuate over time due to inherent uncertainty in the environment. Independent of a volatility parent node
*μ_n*	Mean of belief at node *n*	Mean of the inferred belief at a node. For a volatility node, μ corresponds to a volatility belief
*σ_n*	Precision at node *n*	Precision (1/the variance) of the belief state of the node, i.e., the agents certainty
*λ_n*	Autoconnection strength at node *n*	Influence of the previous state of a node on its prediction of the next state (stability of beliefs)

### Statistical analysis

**Figure 4.**
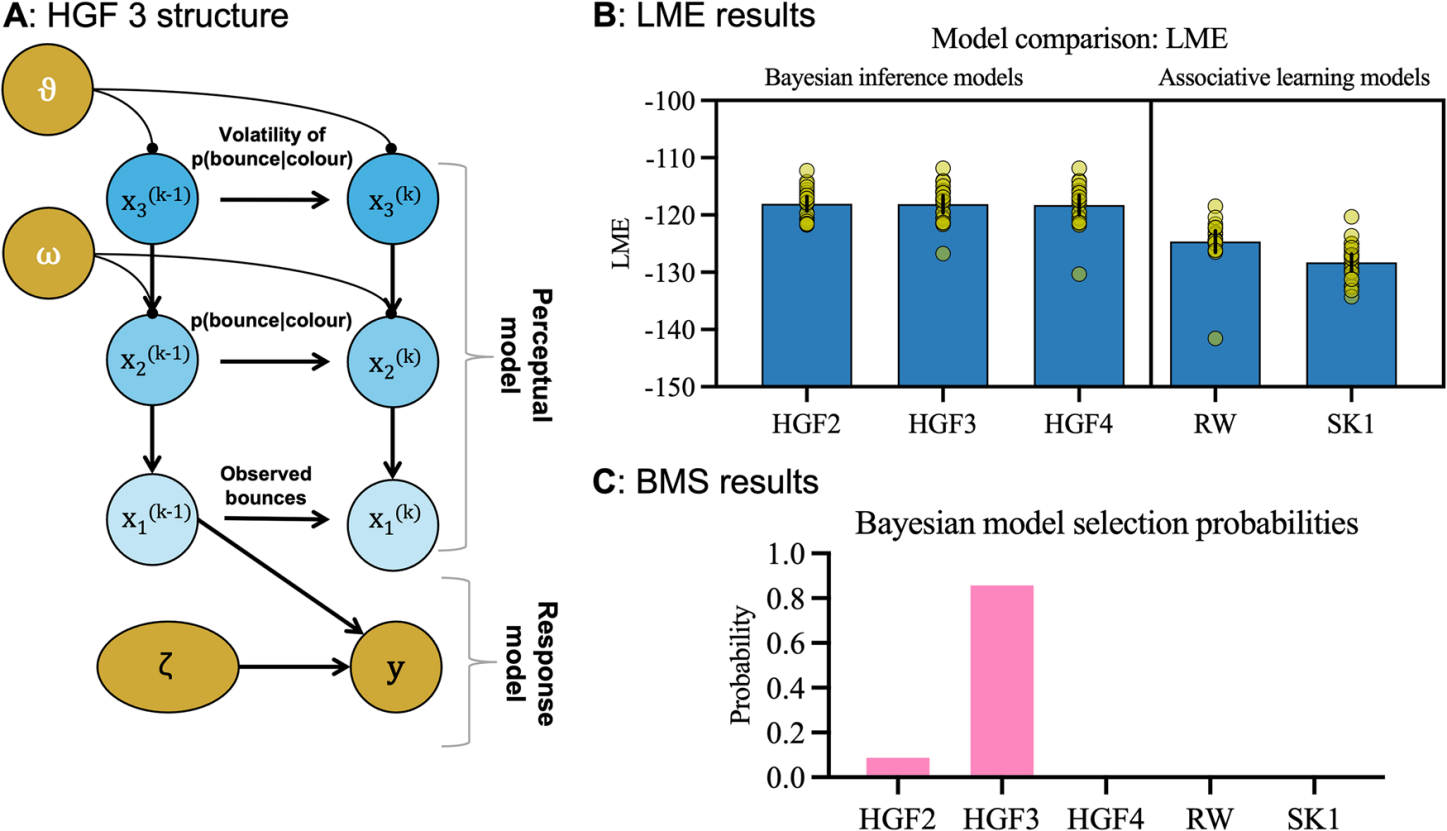
The winning HGF3 model and model comparison results. Panel ***A*** shows the factor graph for the three-level HGF model, illustrating two timepoints, where the lowest level encodes observations, the second level encodes beliefs about outcome probabilities, and the third level encodes beliefs about the rate of change of probabilities (volatility). Panels ***B*** and ***C*** show the results from the model comparison (LME, log model evidence; BMS, Bayesian model selection).

Model comparison was performed using Bayesian model selection (BMS; Rigoux et al., 2014), using spm_BMS.m routines from the SPM12 toolbox (https://www.fil.ion.ucl.ac.uk/spm/software/spm12/) to evaluate the log model evidence (LME) and protected exceedance probabilities of competing models. BMS treats the model as a random variable that could differ between participants, with a fixed (unknown) distribution in the population. It provides an estimate of the probability that a given model outperforms all others in the comparison (the “protected exceedance probability”; Fig. 4).

Subsequent analyses of model parameters were conducted in R (version 2023.09.1) using the brms package (Bürkner, 2017). Bayesian mixed-effect models with weakly informative priors were estimated via Hamiltonian Monte Carlo in Stan, using four chains of 4,000 iterations each (2,000 warm-up). Model convergence was confirmed (all $\hat{R}$ ≈ 1.00, bulk and tail ESS > 1,000). Posterior distributions were summarized using 95% highest density intervals (HDIs), and evidence strength was quantified using Bayes factors calculated via the bayestestR package (Makowski et al., 2019). To examine how individual differences in model-derived parameters related to task performance, we used Bayesian robust regression with a Student's *t* likelihood and standardized predictors and outcomes. See Supplementary File 4, 5, and 6 available on https://osf.io/yq2bv/files for diagnostics.

## Results

### Model comparison

Three versions of the HGF [two-level (HGF2), three-level (HGF3), and four-level (HGF4)] were compared with two associative learning models (R–W and Sutton K1) to determine which best explained observed changes to anticipatory eye movements. As hypothesized, the Bayesian model variants were better able to predict observed behaviors than the two associative learning models (Fig. 4*B*), lending further support for the value of Bayesian models of perception and action. The three HGF models had similar LME scores, but the HGF3 (see Fig. 4*A* for a factor graph) had a much higher protected exceedance probability when using BMS (Fig. 4*C*). The HGF3 was therefore selected as the winning model. Compared with the HGF2, which is functionally equivalent to a Kalman filter, the HGF3 contains an additional node for tracking dynamic changes in environmental volatility. The better performance of the HGF3 suggests that participants inferred the volatility of the environment to enable them to adapt to the frequent shifts in the true associations present across trials (Fig. 2, right). These results therefore suggest that Bayesian inference was the more likely explanation for oculomotor behaviors in this task. To assess parameter identifiability, we conducted a parameter recovery analysis (see Supplementary File 3 available on https://osf.io/yq2bv/files). Here, we simulated 80 datasets with known parameter values, fit the HGF model to each simulated dataset, and then calculated correlations between true and recovered parameters to confirm that key model parameters could be reliably estimated from behavioral data. This revealed an acceptable mean correlation of *r* = 0.83. To further validate our model comparison approach, we performed a model recovery analysis simulating data from each model family (HGF and RL) and testing whether our fitting procedure could correctly identify the generative model. Results showed moderate overall recovery (73.5%) but in an asymmetric pattern: HGF models were correctly recovered in 96% of simulations, while RL models were recovered in just 51% of cases. This asymmetry in model recovery reveals that while we can be confident in detecting HGF when it truly generates the data (96% recovery), the poor RL recovery (51%) indicates that HGF may be identified even when RL is the true model. This reflects a common challenge in computational modeling, where more flexible models can accommodate patterns generated by simpler mechanisms, but the reverse is rare (Palminteri et al., 2017; Wilson and Collins, 2019). Nevertheless, the substantial superiority of HGF over RL models in our empirical data (with high protected exceedance probabilities; Fig. 4*C*) provides strong evidence for hierarchical learning in this task. Importantly, the asymmetric model recovery pattern means that observing such clear preference for HGF is unlikely to reflect mere model flexibility—had our data been generated by simpler RL mechanisms, we would expect more ambiguous model comparison results rather than the decisive evidence we obtained.

### State-switching versus continuous learning

**Figure 5.**
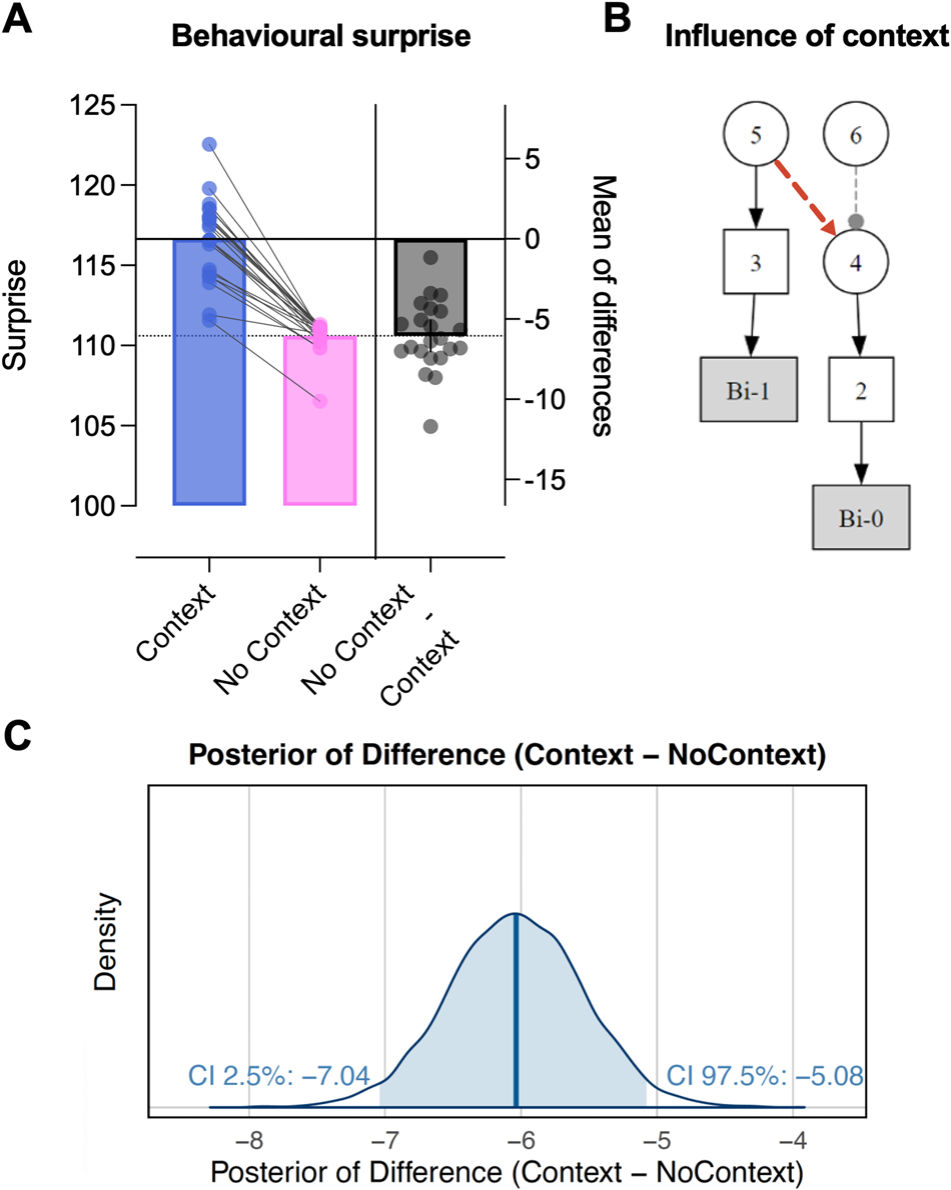
Comparison of surprise of observed behaviors for continuous learning versus state-switching models. Panel ***A*** shows comparison of raw surprise values. Panel ***B*** shows the context switching model structure, with the red dashed arrow indicating the top–down effect that is removed in the No Context model, effectively making behaviors predicted by a simple HGF3. Panel ***C*** shows the CI plot for the difference in model surprise values.

Next, we compared the winning HGF3 model with the more complex state-switching model (Fig. 5*A*,*B*). We conducted Bayesian mixed-effect modeling to estimate the difference in behavioral surprise values between the two models. A Bayesian approach was chosen to allow full quantification of uncertainty in model parameters. The model, supplied with weakly informative priors, was fit using Hamiltonian Monte Carlo via Stan, with four chains of 4,000 iterations each (2,000 warm-up), ensuring convergence ( $\hat{R}$ ≈ 1.00, bulk and tail ESS > 1,000; see also see Supplementary File 4 available on https://osf.io/yq2bv/files for prior–posterior, chain convergence, and posterior predictive check plots). There was strong evidence that surprise ratings were substantially lower for the “No Context” (i.e., HGF3) model compared with the “Context” model [posterior mean = –6.04; 95% HDI [–7.04, –5.08]; see Fig. 5*C* for credible interval (CI) plot]. A Bayes factor was also computed from the fitted model which similarly indicated strong evidence in favor of the No Context model [BF_10_ = 4.89 × 10^8^], indicating that eye movements were better explained by continuous belief updating than state switching. As Layers 2 and 3 of the HGF3 model could be accounting for much of the variance in transitions between probability states (thereby reducing the utility of the context node), we also performed the state-switching versus continuous learning comparison with the volatility tracking node removed. This produced a similar result, with surprise values clearly lower for the continuous learning version (posterior mean = –13.34; 95% HDI [–22.12, –4.16]; BF₁₀ = 25.94; see Supplementary File 7 available on https://osf.io/yq2bv/files).

**Figure 6.**
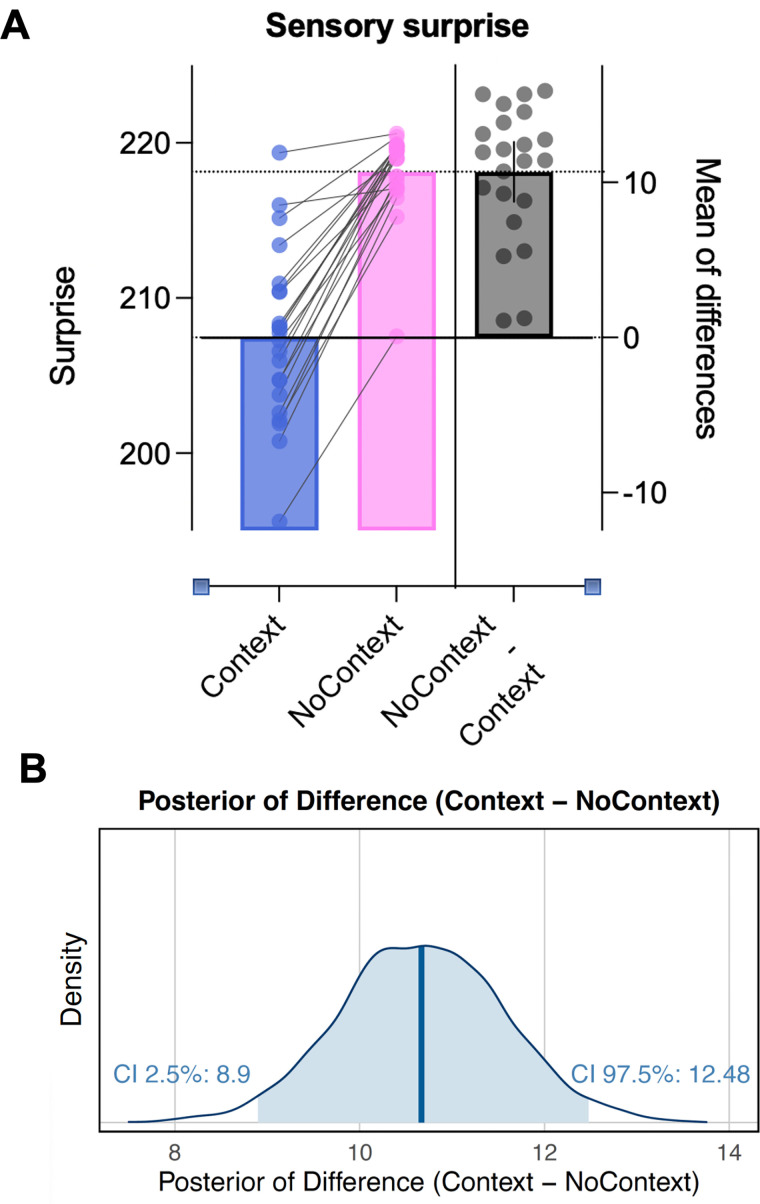
Total surprise of sensory observations with and without a top–down influence of context. Panel ***A*** shows comparison of raw surprise values. Panel ***B*** shows the CI plot for the difference in model surprise values.

Given that participants did not appear to exhibit state-switching dynamics, we investigated whether a state-switching generative model was actually beneficial in this task, at least from a computational perspective. To do this, we computed the surprise of the sensory observations (i.e., observed ball bounces) in light of the trajectory of probabilistic beliefs about color–bounce associations. Specifically, we did this for two versions of the state-switching model; one with a top–down influence of observed context (i.e., the fully connected “Context model”) and one where inferences about color–bounce associations were entirely independent of context (i.e., the unconnected version of the model in Fig. 5*B*). The Bayesian mixed-effect model ( $\hat{R}$ ≈ 1.00, bulk and tail ESS > 1,000; see Supplementary File 5 available on https://osf.io/yq2bv/files for additional plots) indicated strong evidence that the fully connected contextual inference model exhibited lower surprise values when predicting ball bounce outcomes compared with the disconnected no context (i.e., HGF3) model (posterior mean = 10.57; 95% HDI [8.55, 12.58]; see Fig. 6*A* for raw values and Fig. 6*B* for the CI plot). A Bayes factor computed similarly indicated strong evidence in favor of the fully connected model [BF_10_ = 7.53 × 10^8^], indicating it was substantially better at predicting trial outcomes. This result confirms that for the current sensorimotor task, a state-inference model with a top–down influence on eye movements could make better oculomotor predictions but that participants did not spontaneously use this strategy.

### Relationship between task execution and model parameters

Finally, we examined whether the model parameters predicted task performance to understand how individual learning characteristics related to overall execution. We examined the three optimized parameters (*λ*_2, *λ*_4, and *ω*_4) plus the precision of beliefs at Node 4 (*σ*_4) and the volatility belief (*μ*_6; note: we continue to refer to the nodes in the No Context model using the numbering system in Fig. 4*B* for consistency, even though the odd numbered nodes are not part of the model). Overall task performance was indexed by radial error, calculated as the Euclidean distance of the ball from the target center on collision with the wall. We used a Bayesian robust regression model with a Student's *t* likelihood to account for potential non-normality. Weakly informative priors were set, and the model was estimated using four MCMC chains with 4,000 iterations each (2,000 warm-up), yielding 8,000 post-warm-up draws. All variables were standardized prior to analysis, allowing regression coefficients to be interpreted as partial correlations ( $\hat{R}$ ≈ 1.00, bulk and tail ESS > 1,000; see Supplementary File 6 available on https://osf.io/yq2bv/filess for posterior predictive checks). CIs for the regression coefficients and corresponding Bayes factors indicated that none of the predictors showed clear relationships with task performance (Table 3).

**Table 3.** Results of Bayesian regression models predicting performance

DV	Predictor	Estimate	Lower 95% CI	Upper 95% CI	BF_10_
*Overall radial error* ^a^	*λ*_2	0.02	−0.43	0.48	0.25
	*λ*_4	0.06	−0.56	0.71	0.32
	*ω*_4	0.14	−1.30	1.55	0.77
	*μ*_6	0.68	−0.30	1.63	1.43
	*σ*_4	−0.39	−1.79	0.99	0.83
*E-UE radial error* ^a^	*λ*_2	−0.30	−0.71	0.13	0.58
	*λ*_4	0.45	−0.23	1.12	0.85
	*ω*_4	−0.44	−1.94	1.03	0.89
	*μ*_6	0.18	−0.81	1.15	0.53
	*σ*_4	−0.22	−1.63	1.26	0.79

a Estimate represents change in standardized dependent variable per 1 SD increase in predictor (i.e., a standardized regression coefficient).

We also examined whether the individual learning parameters predicted the difference in performance between expected and unexpected trial outcomes. “Expected” and “unexpected” in this context were defined according to the ball color and room color cues. Bounce outcomes consistent with the dominant probability mappings in the sub-block (e.g., a high bounce when the ball was green and room was pink would be classed as “expected” and a low bounce classed as “unexpected”). We subsequently calculated the expected minus unexpected (E − UE) difference for radial errors. A higher difference value would indicate that participants were responding to unexpected ball bounce trajectories less successfully than on expected trials (as indicative of greater behavioral surprise).

## Discussion

The present study examined how observers dynamically infer hidden states to guide their perception and action. This inquiry is grounded in the broader objective of unraveling how hierarchical representations in the brain, particularly in prefrontal and parietal cortices (Badre and D’Esposito, 2009), enable humans to adapt flexibly to ever-changing and uncertain environments (Friston, 2010; Clark, 2015; Yon and Frith, 2021). Such insights are crucial for illuminating how skilled actions—whether in sport, daily life, or clinical contexts—are mediated by the brain's ability to represent and respond to hidden states. In contrast to prior work that has primarily relied on simplified keypress associative tasks (Bartolo and Averbeck, 2020; Zika et al., 2023), we employed a rich, real-world motor task—intercepting a bouncing tennis ball—to investigate learning and inference under naturalistic sensorimotor demands rather than through discrete choice behavior.

Firstly, we found evidence that Bayesian inference models better accounted for the trial-to-trial changes in anticipatory eye movements linked to the learning of color–bounciness associations. This accords with previous evidence that humans combine noisy sensory signals with prior expectations to infer properties such as motion or depth (Kersten et al., 2004; Knill, 2007) and adapt motor and oculomotor movements to account for uncertainty (Körding and Wolpert, 2004; Arthur and Harris, 2021). Our findings extend this literature by showing that such probabilistic learning processes also govern the acquisition of abstract contextual associations—such as linking a color cue to the expected dynamics of an object—and that these hierarchical beliefs are continuously updated as new evidence accumulates. Our results therefore support the view that sensorimotor learning is fundamentally inferential, involving the construction and refinement of internal generative models—likely supported by interactions between sensory cortices and medial prefrontal areas—that guide perception and action under uncertainty (Körding and Wolpert, 2004; Friston et al., 2012; O’Reilly, 2013).

Next, we observed that participants’ anticipatory gaze behavior was better explained by a continuous learning model than by one including inference of a hidden state variable. This finding implies that, in this task, participants did not infer the presence of some higher-order hidden state that was switching over time (either cued by or driven by the room color changes), choosing instead to rely on more gradual belief updating. Unlike more discrete or categorical learning processes (Wilson et al., 2014; Schuck et al., 2016; Zika et al., 2023), the learning of complex sensorimotor tasks may rely more on continuous, probabilistic updating. This distinction could reflect a fundamental difference in the inferential demands of continuous action versus discrete choice. Dynamic motor skills, such as intercepting a bouncing ball, unfold in real time and require the ongoing integration of temporally dense sensory input with fine-grained motor predictions when state boundaries are ambiguous or in flux. In contrast, discrete choice tasks often present well-defined, punctate events and responses, where the inference of latent contextual shifts (e.g., reversals or rule changes) confers a clear adaptive advantage. Accordingly, the absence of abrupt behavioral shifts in our data may reflect the brain's prioritization of smooth, precision-weighted updates in dynamic sensorimotor environments, where the costs of overinterpreting noise as a state change are high and where action must remain fluid and responsive. Therefore, in the context of skilled action, such as in sports or clinical rehabilitation, our findings suggest that the brain may prioritize flexibility and ongoing adaptation over discrete re-evaluation of context.

Despite this evidence that participants were most likely exhibiting continuous learning dynamics rather than hidden state inference, we found that the simulated state-switching model outperformed the continuous learning model in predicting the actual ball bounces (as shown by reduced surprise of the observations under the state-switching model; Fig. 6*A*). This finding raises intriguing questions about the optimality of human learning behaviors. Under naturalistic conditions, humans achieve near optimal performance in many perceptual (Knill and Pouget, 2004), statistical learning (Fiser et al., 2010), motor control (Wolpert and Ghahramani, 2000), and inductive reasoning (Griffiths and Tenenbaum, 2006) tasks. As Bayesian probability calculations are optimal, by definition, this has raised the concern that when humans do not behave optimally that they are, therefore, not Bayesian. However, Bayesian models describe how information is combined probabilistically, but they do not guarantee optimal behavior (Weiss et al., 2002). For example, humans may misestimate priors or likelihoods yet combine that information optimally. Alternatively, constraints like cognitive limits, noisy computations, or biases can make behavior suboptimal. Suboptimality therefore does not necessarily imply a departure from Bayesian reasoning; it may instead reflect how Bayesian inference operates under bounded resources (Lieder and Griffiths, 2020). Our findings may be an illustration of this bounded optimality effect.

Additionally, humans do not just passively learn associations but make causal inferences based on reasoning about plausible causality relations and prior beliefs built from long-term experiences (Glymour, 2001, 2003). If participants did not perceive or expect a meaningful causal relationship between wall color and the bounce dynamics (e.g., unlike a drier cricket pitch plausibly influencing the spin and trajectory of balls), they may have failed to construct the appropriate causal model. Without this structural inference, top–down contextual modulation would not occur. In contrast to studies in sport science that have explicitly informed participants about relevant cues (McRobert et al., 2011; Gredin et al., 2018; Runswick et al., 2018)—thereby priming them to attend to those features—our task relied on implicit learning processes. This design more closely mirrors naturalistic settings, where cue relevance must be inferred rather than instructed. Our findings therefore highlight a critical distinction between possessing the computational tools for Bayesian inference and deploying them effectively under conditions of structural uncertainty and limited causal guidance. Future studies could, therefore, look to examine whether the plausibility of the causal influence of different cues causes more distinct shifts in sensorimotor behavior.

Findings relating to performance did not show strong relationships between model parameters and task success. Across all regression models, the posterior estimates for key learning parameters showed wide CIs overlapping zero, and Bayes factors indicated anecdotal to moderate evidence against any meaningful effects. This suggests that individual differences in learning dynamics—such as the precision of volatility estimates or learning rates—were not robustly associated with how well participants performed overall. This may indicate that the computational variables captured by the model reflect latent learning processes that are only loosely coupled with overt task performance. Alternatively, performance outcomes in this task such as interception success or radial error may have been too coarse to sensitively reflect underlying differences in belief updating or model-based inference. Thus, while the model captured key features of gaze behavior, its parameters may not serve as strong predictors of behavioral proficiency in this kind of task.

Disruptions to the inferential mechanisms examined in this work, potentially involving atypical function in networks spanning the prefrontal cortex, striatum, and cerebellum, have been implicated in clinical conditions such as autism, obsessive–compulsive disorder, and schizophrenia (Fletcher and Frith, 2009; Maia and Frank, 2011; Gillan and Robbins, 2014), where atypical updating or inflexible model selection may underlie characteristic differences in learning, perception, and motor control. While our study was not designed to investigate these conditions, the principles it reveals—such as the trade-off between continuous updating and discrete state inference—may offer a useful framework for understanding the computational basis of atypical behavior. Additionally, the opportunity to study these inference processes using an ecologically valid movement task offers opportunities for exploring how these mechanisms vary across populations, and how interventions might scaffold more adaptive model construction and updating to address the challenges and inequalities faced by neurodivergent people.

### Limitations

As with all computational modeling approaches, our findings come with certain constraints. Although we identified which candidate model best captured the observed data, it 's important to recognize that the true optimal model may lie outside the set we tested. Our models are paramorphic representations of cognitive processes; they approximate underlying mechanisms but cannot fully capture the complexity and nuance of real-world phenomena. Moreover, model selection inevitably involves trade-offs between model complexity and explanatory power, with the possibility that some subtle but critical variables remain unaccounted for. Thus, while our models provide valuable insights into the dynamics of sensorimotor learning, they are simplifications that should be interpreted in the context of these broader uncertainties.

### Conclusion

The present findings contribute to a growing body of work suggesting that sensorimotor learning is governed by probabilistic inference and ongoing belief updating, particularly in dynamic environments where continuous action is required. By showing that participants updated their beliefs in a graded, rather than a categorical or discrete, manner in response to associative contextual cues, our study highlights the role of flexible model tuning in guiding anticipatory behavior. Our findings also highlight the value of the computational approach for understanding underpinning inference processes form eye movements. These insights extend beyond sport and real-world motor tasks; they also speak to fundamental principles of how the brain constructs internal models of the world to guide perception and action.

## Data Availability

All relevant data and code are available online from: https://osf.io/yq2bv/files.

## References

[B3] Arthur T, Harris DJ (2021) Predictive eye movements are adjusted in a Bayes-optimal fashion in response to unexpectedly changing environmental probabilities. Cortex 145:212–225. 10.1016/j.cortex.2021.09.017 34749190

[B1] Arthur T, Harris D, Buckingham G, Brosnan M, Wilson M, Williams G, Vine S (2021) An examination of active inference in autistic adults using immersive virtual reality. Sci Rep 11:20377. 10.1038/s41598-021-99864-y 34645899 PMC8514518

[B2] Arthur T, Vine S, Buckingham G, Brosnan M, Wilson M, Harris D (2023) Testing predictive coding theories of autism spectrum disorder using models of active inference. PLoS Comput Biol 19:e1011473. 10.1371/journal.pcbi.1011473 37695796 PMC10529610

[B4] Badre D, D’Esposito M (2009) Is the rostro-caudal axis of the frontal lobe hierarchical? Nat Rev Neurosci 10:659–669. 10.1038/nrn2667 19672274 PMC3258028

[B5] Bartolo R, Averbeck BB (2020) Prefrontal cortex predicts state switches during reversal learning. Neuron 106:1044–1054.e4. 10.1016/j.neuron.2020.03.024 32315603 PMC7422923

[B6] Behrens TEJ, Woolrich MW, Walton ME, Rushworth MFS (2007) Learning the value of information in an uncertain world. Nat Neurosci 10:1214–1221. 10.1038/nn1954 17676057

[B7] Berniker M, Voss M, Kording K (2010) Learning priors for Bayesian computations in the nervous system. PLoS One 5:e12686. 10.1371/journal.pone.0012686 20844766 PMC2937037

[B8] Bürkner P-C (2017) brms: an R package for Bayesian multilevel models using Stan. J Stat Softw 80:1–28. 10.18637/jss.v080.i01

[B9] Cesqui B, van de Langenberg R, Lacquaniti F, d’Avella A (2013) A novel method for measuring gaze orientation in space in unrestrained head conditions. J Vis 13:28. 10.1167/13.8.28 23902754

[B10] Cisek P, Kalaska JF (2010) Neural mechanisms for interacting with a world full of action choices. Annu Rev Neurosci 33:269–298. 10.1146/annurev.neuro.051508.135409 20345247

[B11] Clark A (2015) Surfing uncertainty: prediction, action, and the embodied mind. Oxford: Oxford University Press.

[B12] Diaz G, Cooper J, Hayhoe M (2013a) Memory and prediction in natural gaze control. Philos Trans R Soc Lond B Biol Sci 368:20130064. 10.1098/rstb.2013.0064 24018726 PMC3758207

[B13] Diaz G, Cooper J, Rothkopf C, Hayhoe M (2013b) Saccades to future ball location reveal memory-based prediction in a virtual-reality interception task. J Vis 13:20. 10.1167/13.1.20 PMC3587002 23325347

[B14] Felleman DJ, Van Essen DC (1991) Distributed hierarchical processing in the primate cerebral cortex. Cereb Cortex 1:1–47. 10.1093/cercor/1.1.1 1822724

[B15] Fiser J, Berkes P, Orbán G, Lengyel M (2010) Statistically optimal perception and learning: from behavior to neural representations. Trends Cogn Sci 14:119–130. 10.1016/j.tics.2010.01.003 20153683 PMC2939867

[B16] Fletcher PC, Frith CD (2009) Perceiving is believing: a Bayesian approach to explaining the positive symptoms of schizophrenia. Nat Rev Neurosci 10:48–58. 10.1038/nrn2536 19050712

[B18] Fooken J, Spering M (2020) Eye movements as a readout of sensorimotor decision processes. J Neurophysiol 123:1439–1447. 10.1152/jn.00622.2019 32159423 PMC7191514

[B17] Fooken J, Baltaretu BR, Barany DA, Diaz G, Semrau JA, Singh T, Crawford JD (2023) Perceptual-cognitive integration for goal-directed action in naturalistic environments. J Neurosci 43:7511–7522. 10.1523/JNEUROSCI.1373-23.2023 37940592 PMC10634571

[B19] Frässle S, et al. (2021) TAPAS: an open-source software package for translational neuromodeling and computational psychiatry. Neuroscience. Available at: http://biorxiv.org/lookup/doi/10.1101/2021.03.12.435091 [Accessed Dec. 28, 2021].

[B20] Friston K (2010) The free-energy principle: a unified brain theory? Nat Rev Neurosci 11:127–138. 10.1038/nrn2787 20068583

[B21] Friston K, Adams R, Perrinet L, Breakspear M (2012) Perceptions as hypotheses: saccades as experiments. Front Psychol 3:151. 10.3389/fpsyg.2012.00151 22654776 PMC3361132

[B22] Friston K, FitzGerald T, Rigoli F, Schwartenbeck P, O'Doherty J, Pezzulo G (2016) Active inference and learning. Neurosci Biobehav Rev 68:862–879. 10.1016/j.neubiorev.2016.06.022 27375276 PMC5167251

[B23] Friston KJ, Daunizeau J, Kiebel SJ (2009) Reinforcement learning or active inference? PLoS One 4:e6421. 10.1371/journal.pone.0006421 19641614 PMC2713351

[B24] Gillan CM, Robbins TW (2014) Goal-directed learning and obsessive–compulsive disorder. Philos Trans R Soc Lond B Biol Sci 369:20130475. 10.1098/rstb.2013.0475 25267818 PMC4186229

[B25] Glymour C (2003) Learning, prediction and causal Bayes nets. Trends Cogn Sci 7:43–48. 10.1016/S1364-6613(02)00009-8 12517358

[B26] Glymour CN (2001) The mind’s arrows: Bayes nets and graphical causal models in psychology. Boston: MIT Press.

[B27] Gredin NV, Bishop DT, Broadbent DP, Tucker A, Williams AM (2018) Experts integrate explicit contextual priors and environmental information to improve anticipation efficiency. J Exp Psychol Appl 24:509–520. 10.1037/xap0000174 30024211

[B28] Griffiths TL, Tenenbaum JB (2006) Optimal predictions in everyday cognition. Psychol Sci 17:767–773. 10.1111/j.1467-9280.2006.01780.x 16984293

[B29] Harris D, Vine S, Wilson M, Arthur T (2023) The relationship between environmental statistics and predictive gaze behaviour during a manual interception task: eye movements as active inference. Comput Brain Behav 7:225–241. 10.1007/s42113-023-00190-5 PMC13298623 42460434

[B30] Harris DJ, Arthur T, Vine SJ, Liu J, Abd Rahman HR, Han F, Wilson MR (2022) Task-evoked pupillary responses track precision-weighted prediction errors and learning rate during interceptive visuomotor actions. Sci Rep 12:22098. 10.1038/s41598-022-26544-w 36543845 PMC9772236

[B31] Harris DJ, Arthur T, Vine SJ, Rahman HRA, Liu J, Han F, Wilson MR (2023) The effect of performance pressure and error-feedback on anxiety and performance in an interceptive task. Front Psychol 14:1182269. 10.3389/fpsyg.2023.1182269 37251048 PMC10215563

[B32] Heilbron M, Meyniel F (2019) Confidence resets reveal hierarchical adaptive learning in humans. PLoS Comput Biol 15:e1006972. 10.1371/journal.pcbi.1006972 30964861 PMC6474633

[B33] Hipólito I, Kirchhoff M (2023) Breaking boundaries: the Bayesian brain hypothesis for perception and prediction. Conscious Cogn 111:103510. 10.1016/j.concog.2023.103510 37058949

[B34] Iglesias S, Mathys C, Brodersen KH, Kasper L, Piccirelli M, den Ouden HEM, Stephan KE (2013) Hierarchical prediction errors in midbrain and basal forebrain during sensory learning. Neuron 80:519–530. 10.1016/j.neuron.2013.09.009 24139048

[B35] Kass RE, Raftery AE (1995) Bayes factors. J Am Stat Assoc 90:773–795. 10.1080/01621459.1995.10476572

[B36] Kersten D, Mamassian P, Yuille A (2004) Object perception as Bayesian inference. Annu Rev Psychol 55:271–304. 10.1146/annurev.psych.55.090902.142005 14744217

[B37] Knill DC (2007) Learning Bayesian priors for depth perception. J Vis 7:13. 10.1167/7.8.13 17685820

[B38] Knill DC, Pouget A (2004) The Bayesian brain: the role of uncertainty in neural coding and computation. Trends Neurosci 27:712–719. 10.1016/j.tins.2004.10.007 15541511

[B39] Körding KP, Wolpert DM (2004) Bayesian integration in sensorimotor learning. Nature 427:244–247. 10.1038/nature02169 14724638

[B40] Krassanakis V, Filippakopoulou V, Nakos B (2014) EyeMMV toolbox: an eye movement post-analysis tool based on a two-step spatial dispersion threshold for fixation identification. J Eye Mov Res 7:1–10. 10.16910/jemr.7.1.1

[B41] Legrand N, Weber L, Waade PT, Daugaard AHM, Khodadadi M, Mikuš N, Mathys C (2024) pyhgf: a neural network library for predictive coding. Available at: http://arxiv.org/abs/2410.09206 [Accessed Nov. 15, 2024].

[B42] Lieder F, Griffiths TL (2020) Resource-rational analysis: understanding human cognition as the optimal use of limited computational resources. Behav Brain Sci 43:e1. 10.1017/S0140525X1900061X 30714890

[B43] Maia TV, Frank MJ (2011) From reinforcement learning models to psychiatric and neurological disorders. Nat Neurosci 14:154–162. 10.1038/nn.2723 21270784 PMC4408000

[B44] Makowski D, Ben-Shachar M, Lüdecke D (2019) Bayestestr: describing effects and their uncertainty, existence and significance within the Bayesian framework. J Open Source Softw 4:1541. 10.21105/joss.01541

[B45] Mann DL, Nakamoto H, Logt N, Sikkink L, Brenner E (2019) Predictive eye movements when hitting a bouncing ball. J Vis 19:28. 10.1167/19.14.28 31891654

[B46] Mathys CD, Daunizeau J, Friston K, Stephan K (2011) A Bayesian foundation for individual learning under uncertainty. Front Hum Neurosci 5:39. 10.3389/fnhum.2011.00039 21629826 PMC3096853

[B47] Mathys CD, Lomakina EI, Daunizeau J, Iglesias S, Brodersen KH, Friston KJ, Stephan KE (2014) Uncertainty in perception and the hierarchical Gaussian filter. Front Hum Neurosci 8:825. 10.3389/fnhum.2014.00825 25477800 PMC4237059

[B48] McRobert AP, Ward P, Eccles DW, Williams AM (2011) The effect of manipulating context-specific information on perceptual–cognitive processes during a simulated anticipation task. Br J Psychol 102:519–534. 10.1111/j.2044-8295.2010.02013.x 21752003

[B49] Niehorster DC, Li L, Lappe M (2017) The accuracy and precision of position and orientation tracking in the HTC vive virtual reality system for scientific research. i-Perception 8:2041669517708205. 10.1177/2041669517708205 28567271 PMC5439658

[B50] O’Reilly JX (2013) Making predictions in a changing world—inference, uncertainty, and learning. Front Neurosci 7:105. 10.3389/fnins.2013.00105 23785310 PMC3682109

[B51] Palminteri S, Wyart V, Koechlin E (2017) The importance of falsification in computational cognitive modeling. Trends Cogn Sci 21:425–433. 10.1016/j.tics.2017.03.011 28476348

[B52] Patil A, Huard D, Fonnesbeck CJ (2010) PyMC: Bayesian stochastic modelling in python. J Stat Softw 35:1. 10.18637/jss.v035.i04 21603108 PMC3097064

[B53] Rescorla RA, Wagner AR (1972) Classical conditioning II: current research and theory. In: Classical conditioning II: current research and theory (Black AH, Prokasy WF, eds), pp 64–99. New York: Appleton-Century Crofts.

[B54] Rigoux L, Stephan KE, Friston KJ, Daunizeau J (2014) Bayesian model selection for group studies—revisited. Neuroimage 84:971–985. 10.1016/j.neuroimage.2013.08.065 24018303

[B55] Runswick OR, Roca A, Williams AM, Bezodis NE, Mcrobert AP, North JS (2018) The impact of contextual information and a secondary task on anticipation performance: an interpretation using cognitive load theory. Appl Cogn Psychol 32:141–149. 10.1002/acp.3386

[B56] Salvucci DD, Goldberg JH (2000) Identifying fixations and saccades in eye-tracking protocols. In: *Proceedings of the symposium on eye tracking research & applications - ETRA ‘00*, pp 71–78. Palm Beach Gardens, Florida, United States: ACM Press.

[B57] Schönbrodt FD, Wagenmakers E-J (2018) Bayes factor design analysis: planning for compelling evidence. Psychon Bull Rev 25:128–142. 10.3758/s13423-017-1230-y 28251595

[B58] Schuck NW, Cai MB, Wilson RC, Niv Y (2016) Human orbitofrontal cortex represents a cognitive map of state space. Neuron 91:1402–1412. 10.1016/j.neuron.2016.08.019 27657452 PMC5044873

[B59] Smith R, Friston KJ, Whyte CJ (2022) A step-by-step tutorial on active inference and its application to empirical data. J Math Psychol 107:102632. 10.1016/j.jmp.2021.102632 35340847 PMC8956124

[B60] Sutton RS (1992) Gain adaptation beats least squares? In: *Proceedings of the 7th Yale workshop on adaptive and learning systems*, Vol 161, pp 166.

[B61] Tzovara A, Korn CW, Bach DR (2018) Human Pavlovian fear conditioning conforms to probabilistic learning. PLoS Comput Biol 14:e1006243. 10.1371/journal.pcbi.1006243 30169519 PMC6118355

[B62] Vaidya AR, Jones HM, Castillo J, Badre D (2021) Neural representation of abstract task structure during generalization (Liljeholm M, Ivry RB, Ranganath C, Michelmann S, eds). Elife 10:e63226. 10.7554/eLife.63226 33729156 PMC8016482

[B63] Weber LA, Waade PT, Legrand N, Møller AH, Stephan KE, Mathys C (2023) The generalized hierarchical Gaussian filter. Available at: http://arxiv.org/abs/2305.10937 [Accessed July 11, 2023].

[B64] Weiss Y, Simoncelli EP, Adelson EH (2002) Motion illusions as optimal percepts. Nat Neurosci 5:598–604. 10.1038/nn0602-858 12021763

[B66] Wilson RC, Collins AG (2019) Ten simple rules for the computational modeling of behavioral data (Behrens TE, ed). Elife 8:e49547. 10.7554/eLife.49547 31769410 PMC6879303

[B65] Wilson RC, Takahashi YK, Schoenbaum G, Niv Y (2014) Orbitofrontal cortex as a cognitive map of task space. Neuron 81:267–279. 10.1016/j.neuron.2013.11.005 24462094 PMC4001869

[B67] Wolpert DM, Ghahramani Z (2000) Computational principles of movement neuroscience. Nat Neurosci 3:1212–1217. 10.1038/81497 11127840

[B68] Yon D, Frith CD (2021) Precision and the Bayesian brain. Curr Biol 31:R1026–R1032. 10.1016/j.cub.2021.07.044 34520708

[B69] Zika O, Wiech K, Reinecke A, Browning M, Schuck NW (2023) Trait anxiety is associated with hidden state inference during aversive reversal learning. Nat Commun 14:4203. 10.1038/s41467-023-39825-3 37452030 PMC10349120

